# Supplementary material for: Identification of photoperiod-induced specific miRNAs in the adrenal glands of Sunite sheep (Ovis aries)
Source: Front Vet Sci. 2022 Jul 22;9:888207. doi: 10.3389/fvets.2022.888207 (PMC9354845; doi:10.3389/fvets.2022.888207)
Supplement: Supplementary file 1 [file Data_Sheet_1.ZIP › Supplementary file/miRDeep2 file.zip!/miRDeep2 file/Novel_Structure/LP42A3/Novel_430.pdf]

Provisional ID : NC_019479.2_21503
Score total : 1.8
Score for star read(s) : -1.3
Score for read counts : 0
Score for mfe : 1.5
Score for randfold : 1.6
Score for cons. seed :
Total read count : 134
Mature read count : 134
Loop read count : 0
Star read count : 0

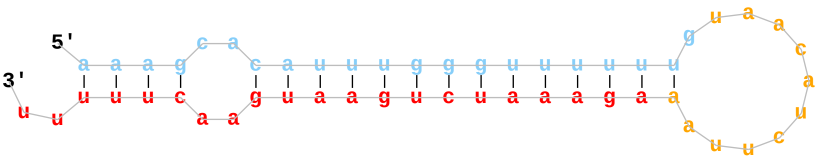

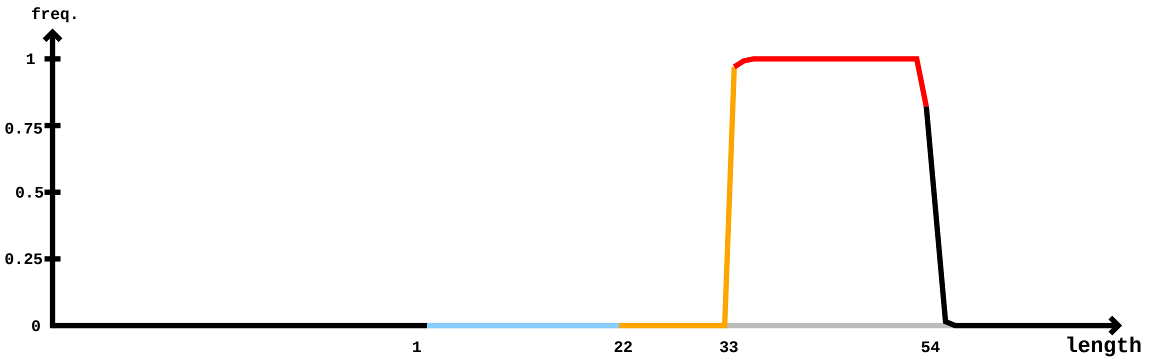

Star Mature

```
5'- accaaaauuacagaggcuaauuuauuggguuggcuaaaaagcacauuuggguuuuuuguaacaucuuaaagaaaucugaaugaacuuuuugggcaacccaauacauucu   -3'    exp
    ......................((((((((((.((((((((..((((((((((((((...........))))))))))))))..)))))))).))))))))))......     reads    mm    sample
    .....................................................................aAaaaucugaaugaacuuuu....................     23       1     seq
    .....................................................................aAaaaucugaaugaacuuuuu...................     65       1     seq
    .....................................................................aAaaaucugaaugaacuuuuug..................     42       1     seq
    ......................................................................gaaaucugaaugaacuuuu....................     1        0     seq
    ......................................................................gaaaCcugaaugaacuuuuug..................     1        1     seq
    ......................................................................gaaaucugaaugaacuuuuugg.................     1        0     seq
    .......................................................................aaaucugaaugaacuuuuugg.................     1        0     seq
```
